# Supplementary material for: High-speed single-molecule imaging reveals signal transduction by induced transbilayer raft phases
Source: J Cell Biol. 2020 Oct 14;219(12):e202006125. doi: 10.1083/jcb.202006125 (PMC7563750; doi:10.1083/jcb.202006125)
Supplement: Table S1 — summarizes colocalization lifetimes (τ1, τ2) and statistical parameters for recruitment of cytoplasmic lipid-anchored molecules at CD59 clusters located in the outer leaflet. [file JCB_202006125_TableS1.docx]

Table S1. Summary of colocalization lifetimes (τ_1_, τ_2_) and statistical parameters for recruitment of cytoplasmic lipid-anchored molecules at CD59 clusters located in outer leaflet

| **CD59 clusters or non–cross-linked CD59** | **Cytoplasmic molecules** | **τ_1_a (ms) (%)** | **τ_2_ (ms) (%)** | **P values** | **No. for correct overlay** | **No. for shifted overlay** |
| --- | --- | --- | --- | --- | --- | --- |
| Clusters | Lyn-FG | 15 ± 0.93 (74) | 80 ± 25 (26) | 0.00076 | 245 | 156 |
| Non–cross linked | Lyn-FG | 19 ± 1.3 (100) | None | 0.86 | 122 | 83 |
| Clusters | Myrpal-N20Lyn-GFP | 20 ± 1.2 (78) | 66 ± 58 (22) | 0.025 | 210 | 127 |
| Clusters | TM-Lyn-GFP | 17 ± 0.92 (100) | None | 0.46 | 93 | 127 |
| Clusters | Palpal-N16GAP43-GFP | 14 ± 1.1 (76) | 71 ± 36 (24) | 0.0023 | 164 | 115 |
| Clusters | GFP-C5Rho-gerger | 10 ± 0.98 (100) | None | 0.97 | 91 | 69 |
| Clusters | FGH-Ras | 27 ± 3.3 (73) | 91 ± 80 (27) | 0.029 | 173 | 90 |
| Non–cross linked | FGH-Ras | 25 ± 1.3 (100) | None | 0.52 | 126 | 83 |
| Clusters + MβCD | FGH-Ras | 18 ± 0.78 (100) | None | 0.41 | 536 | 468 |
| Clusters | GFP-tH | 15 ± 1.1 (53) | 75 ± 53 (47) | 0.072 | 123 | 83 |

The mean and SEM for τ_1_ were determined by fitting the histogram *h*(incidental-by-shift) with a single exponential decay function. The histogram for the correct overlay was fitted with the sum of two exponential decay functions in which the decay time of an exponential function was fixed at τ_1_ determined for *h*(incidental-by-shift). The fractions for the two components were calculated using τ_1_ and three best-fit, free-fitting parameters (two preexponential factors and τ_2_).
