# Supplementary material for: High-speed single-molecule imaging reveals signal transduction by induced transbilayer raft phases
Source: J Cell Biol. 2020 Oct 14;219(12):e202006125. doi: 10.1083/jcb.202006125 (PMC7563750; doi:10.1083/jcb.202006125)
Supplement: Table S2 — summarizes the colocalization lifetimes (τ1, τ2) and statistical parameters for recruitment of cytoplasmic lipid-anchored molecules at Ab-CTXB-GM1 clusters located in the outer leaflet, as compared with results at CTXB-5-GM1 and DNP-DOPE clusters. [file JCB_202006125_TableS2.docx]

Table S2. Summary of colocalization lifetimes (τ_1_, τ_2_) and statistical parameters for recruitment of cytoplasmic lipid-anchored molecules at Ab-CTXB-GM1 clusters located in outer leaflet, as compared with results at CTXB-5-GM1 and DNP-DOPE clusters

| **Outer-leaflet clusters** | **Cytoplasmic molecules** | **τ_1_a (ms) (%)** | **τ_2_ (ms) (%)** | **P values** | **No. for correct overlay** | **No. for shifted overlay** |
| --- | --- | --- | --- | --- | --- | --- |
| Ab-CTXB-GM1 clusters | Lyn-FG | 19 ± 0.79 (73) | 110 ± 32 (27) | 0.013 | 408 | 240 |
| CTXB-5-GM1 | Lyn-FG | 21 ± 1.6 (100) | None | 0.24 | 309 | 165 |
| Ab-CTXB-GM1 clusters | FGH-Ras | 20 ± 1.2 (86) | 97 ± 44 (14) | 0.025 | 160 | 109 |
| CTXB-5-GM1 | FGH-Ras | 34 ± 4.5 (100) | None | 0.52 | 60 | 31 |
| Ab-CTXB-GM1 clusters + MβCD | FGH-Ras | 26 ± 3.5 (100) | None | 0.96 | 67 | 68 |
| DNP-DOPE clusters | FGH-Ras | 21 ± 1.5 (100) | None | 0.39 | 86 | 81 |

^a^The mean and SEM for Ƭ1 were determined by fitting the histogram h(incidental-by-shift) with a single exponential decay function. The histogram for the correct overlay was fitted with the sum of two exponential decay functions in which the decay time of an exponential function was fixed at Ƭ1 determined for h(incidental-by-shift). The fractions for the two components were calculated using Ƭ1 and three best-fit, free-fitting parameters (two preexponential factors and Ƭ2).
