# Supplementary material for: High-speed single-molecule imaging reveals signal transduction by induced transbilayer raft phases
Source: J Cell Biol. 2020 Oct 14;219(12):e202006125. doi: 10.1083/jcb.202006125 (PMC7563750; doi:10.1083/jcb.202006125)
Supplement: Table S3 — summarizes colocalization lifetimes (τ1, τ2) and statistical parameters for the recruitment of the outer-leaflet molecules CD59 and CTXB-5-GM1, at the artificially induced oligomers of cytoplasmic lipid-anchored signaling molecules in the inner leaflet. [file JCB_202006125_TableS3.docx]

Table S3. Summary of colocalization lifetimes (τ_1_, τ_2_) and statistical parameters for the recruitment of the outer-leaflet molecules, CD59 and CTXB-5-GM1, at the artificially induced oligomers of cytoplasmic lipid-anchored signaling molecules in the inner leaflet

| **Outer-leaflet molecules** | **AP20187-Induced clusters of cytoplasmic molecules** | **τ_1_a (ms) (%)** | **τ_2_ (ms) (%)** | **P values** | **No. for correct overlay** | **No. for shifted overlay** |
| --- | --- | --- | --- | --- | --- | --- |
| Non–cross-linked CD59 | Lyn-FG | 17 ± 1.3 (100) | None | 0.18 | 91 | 82 |
| Non–cross-linked CD59 | FGH-Ras | 24 ± 1.1 (100) | None | 0.90 | 139 | 143 |
| CTXB-5-GM1 | Lyn-FG | 22 ± 1.5 (100) | None | 0.071 | 153 | 120 |
| CTXB-5-GM1 | FGH-Ras | 21 ± 1.9 (100) | None | 1.00 | 63 | 43 |

^a^The mean and SEM for Ƭ1 were determined by fitting the histogram h(incidental-by-shift) with a single exponential decay function. The histogram for the correct overlay was fitted with the sum of two exponential decay functions in which the decay time of an exponential function was fixed at Ƭ1 determined for h(incidental-by-shift). The fractions for the two components were calculated using Ƭ1 and three best-fit, free-fitting parameters (two preexponential factors and Ƭ2).
